# A Plea for Higher Education

**Published:** 1890-09

**Authors:** W. D. Hardman

**Affiliations:** Harmony Grove, Ga.


### A PLEA FOR HIGHER MEDICAL EDUCATION.

By W. D. HARDMAN, M. D., HARMONY GROVE, GA.

MR. EDITOR—Recently the General Medical Council of England has been considering the question of an increase in the time devoted to a medical education. Previously the time required in completing their graded course in medicine, including the time spent on the preparatory branches of chemistry, physics, botany, etc., has been four years. Their idea now is to increase this time in order to make their course more thorough, and to keep pace with the German universities. Naturally such steps abroad should turn our minds to our own medical colleges; and when we examine them closely we are compelled to see, as a rule, how meagre and incomplete they are.

A short *résumé* of our system would be in order. We have now about one hundred and twenty medical institutions having the power to confer the degree of M. D. Now it is an alarming fact that quite a number of these institutions are of such a low grade that no civilized country should tolerate them. Yet they exist and thrive as the green bay tree. In fact there is a class of men entering our profession every year, I am sorry to say, who single out such institutions above all the rest. And why should they not? As the degree of M. D. is required in most States to entitle one to practice medicine, and as there will always be more or less quacks and charlatans in our profession until the medical laws are made more stringent, why should they not single out the iustitutions which offer them the best inducements, viz., the shortest term, the smallest fee and the easiest examination.

But let us come to a class which includes the greater part of our medical colleges. These are schools, which scattered all over the United States from Maine to Oregon, and from the lakes to the gulf, are entitled to respectability, but which every fair minded man must admit are far—far below the standard they should maintain. These institutions require a two years' course. The term is, on an average, from twelve to twenty weeks. They require no entrance examination whatever. Any man who can write his name well enough to matriculate is received; and it is no exaggeration when I say that many do matriculate who are totally unable to construct a correct English sentence, and I believe I have seen some who actually could not spell correctly their own post-office. Such men pass on through our colleges into our profession, for the majority of our institutions are too generous to allow an applicant to fail to get a diploma. Few will deny that the above statements are correct, and fewer still of our unbiased men will claim that such a state of affairs should continue.

There is no use to deny that many of the graduates from our ordinary medical colleges make some of our best and brightest practitioners; but with what disaster to their early patients we will leave the reader to imagine. Advocates of these short term schools often point with pleasure to eminent men turned out by these schools, and even compare them with inferior men who had superior medical advantages. But this is no argument in favor of inferior medical training. They point an exception as if it were the rule. Usually men who rise to great distinction in the profession and claim some of the smaller schools as their alma mater, do not stop here, but receive medical training at other superior institutions. It is contrary to all rules of reasoning that the shorter time and the less a man studies the more he will know. It is said of Græfe that he ruined a hat full of eyes before he performed the cataract operation successfully. But he was venturing in an unexplored field, and there is more excuse for mistakes under such circumstances. But what excuse have our law-makers and our medical colleges to render for turning out medical students to whom the greater part of the whole realm of known medicine is still an "unexplored field." I make the above statement because I believe that no fair minded man will for a moment maintain that two courses of four months each gives even the brightest student time to get more than a general smattering of what he should know—especially when many of the institutions in this class admit students with but few questions as to how long they have studied under preceptors.

The above is written without referrence to any special college, for the majority of our institutions come under this class, and it is the standard in the mass that should be raised, and not simply special institutions.

But how is this to be done? Will the medical faculties and trustees of our colleges ever become so awakened as to raise the standard themselves? Even if the majority should become so interested as to do so, would it be entirely successful? We think not. The majority of our colleges might go along and lengthen their term, increase their facilities, demand rigid entrance examinations, and far more comprehensive final examinations, and yet, unless this matter was taken in hand by our legislative bodies there would still remain enough inferior institutions to catch a great number of students who enter the profession to get their degree as cheaply and in as short a time as possible. I do not wish to be understood as saying that there is no need of individual colleges raising their standard until all are forced to do so by law. Great good can and is being done in this direction by a few colleges. Among these are Harvard University, the College of Physicians and Surgeons, New York, the Medico Chirurgical College, Philadelphia, and others. These institutions have already extended their term to three years, and lengthened each term to eight or nine months, and are noted for the excellency of their faculties and the high standard of their curriculum.

But the point I am after is this, how can we bring all of our colleges up to this standard, or an even higher, for as excellent as these named institutions are, there is still room for improvement. We answer, it must be done in the halls of our legislatures and in the halls of congress. A great deal has been said in our journals on this subject recently, and the better part of our profession is ripe for the change. But our journals do not reach the laity and those who make our laws, and they are more or less uninterested. Would it not then be advisable for some of our representative men, or some one appointed by the medical association in each State to write a series of articles in our daily papers and literary magazines instructing the laity on these points, and showing the necessity of such changes; for when the people once see the importance and need of a thing, then our representatives are awakened to action. But some one may say what have the people to do with our medical business? They have much to do with it, for they are the ones most directly concerned.

Appoint committees to appear before our legislature assembled and get them thoroughly aroused on the subject. If we go about it in this way and get more stringent laws, we must of necessity then shut off a great many quacks, and the indifferent class that now crowd our profession. Just how or what those laws should be I do not want to suggest, but I do earnestly hope that we will not let the matter rest until medical training in the United States is brought to a higher standard, and until our sister nations shall not fail to recognize our best schools, because of the mass of inferior ones of which we now boast (?).

